# Incisional hernias following open gynecological surgery: a population-based study

**DOI:** 10.1007/s00404-019-05069-0

**Published:** 2019-03-25

**Authors:** Kerstin Bewö, Johanna Österberg, Mats Löfgren, Gabriel Sandblom

**Affiliations:** 1 Department of Surgery, Mora Hospital, 792 85 Mora, Sweden; 2 Department of Clinical Sciences, Intervention and Technology (CLINTEC), Karolinska Institute, Solna, Sweden; 3 Department of Gynecology, University Hospital of Norrland, Umeå, Sweden; 4 Department of Clinical Science and Education Södersjukhuset, Karolinska Institute, Stockholm, Sweden; 5 Department of Surgery, Södersjukhuset, Stockholm, Sweden

**Keywords:** Incisional hernia, Incidence, Risk factors, Gynecological surgical procedures, Midline incision, Pfannenstiel incision

## Abstract

**Introduction:**

Incisional hernia is a common and costly complication following abdominal surgery. The incidence of incisional hernia after gynecological surgery is not as well studied as that after general surgery.

**Materials and methods:**

The Swedish National Quality Register for Gynecological Surgery (GynOp) collects preoperative, intraoperative, and postoperative information regarding gynecological surgery. Data were extracted from 2006 to 2014. The National Patient Register (NPR) contains physicians’ data from both public and private hospitals. Univariate and multivariate Cox proportional hazard analyzes were performed on risk factors.

**Results:**

Between 2006 and 2014, 39,312 women undergoing open surgery were registered in GynOp. The NPR recorded 526 patients who were diagnosed with or had undergone surgery for incisional hernia. The mean follow-up was 2.8 years. Five years after surgery the cumulative incidence of incisional hernias was 2.0% (95% confidence interval 1.8–2.2%). In multivariate Cox proportional hazard analysis obesity (BMI > 30), age > 60 years, midline incision, smoking, kidney, liver, and pulmonary disease were found to predict an increased risk for incisional hernias (all *p* < 0.05).

**Conclusions:**

There is much to be gained if the patient can cease smoking and lose weight before undergoing abdominal surgery. The Pfannenstiel incision results in fewer incisional hernias and should be considered whenever possible.

## Introduction

Of patients who undergo abdominal surgery an estimated 5–20% develop an incisional hernia. Each year approximately 2500 people in Sweden undergo incisional hernia repair and it represents one of the most common complications after abdominal surgery [1]. Incisional hernias cause a great deal of morbidity and are very costly. Although the techniques for repairing incisional hernias have improved over the last two decades, preventing the forming of incisional hernias may be much more cost-effective than treating them once they become manifest. Poulose et al. [2] have calculated that every 1% reduction in hernia recurrence would result in a US $32 million yearly saving in procedural costs alone. Surgical technique, wound infection, age, smoking, and obesity are known risk factors. Israelsson et al. have performed a number of studies on surgical techniques for closing a midline incision to prevent dehiscence and incisional hernias [3–9].

The incidence of incisional hernias after gynecological surgery is not as well studied as that for gastrointestinal surgery. Since gynecologists rarely operate incisional hernias and they may develop several years after the primary procedure there is little awareness of this problem. Furthermore, the introduction of new surgical techniques for closure of the abdominal wall do not diffuse as rapidly among gynecologists and obstetricians as among general surgeons. The Pfannenstiel incision is associated with a lower incidence of incisional hernias (0–2%) than the midline incision [10, 11]. Nevertheless, a large proportion of gynecological operations are performed using a midline incision due to the need for more extensive access to the abdominal cavity in, e.g., oncological surgery. Franchi et al. [12] reported an incidence of incisional hernias of 16.9% after extended abdominal hysterectomy with bilateral salpingo-oophorectomy. They examined their patients regularly during a follow-up period of ten years and recorded any incisional hernia exceeding 3 cm.

The aim of our study was to determine the incidence of incisional hernia and to identify risk factors associated with incisional hernia development in women undergoing open gynecological surgery.

## Materials and methods

**Fig. 1:**
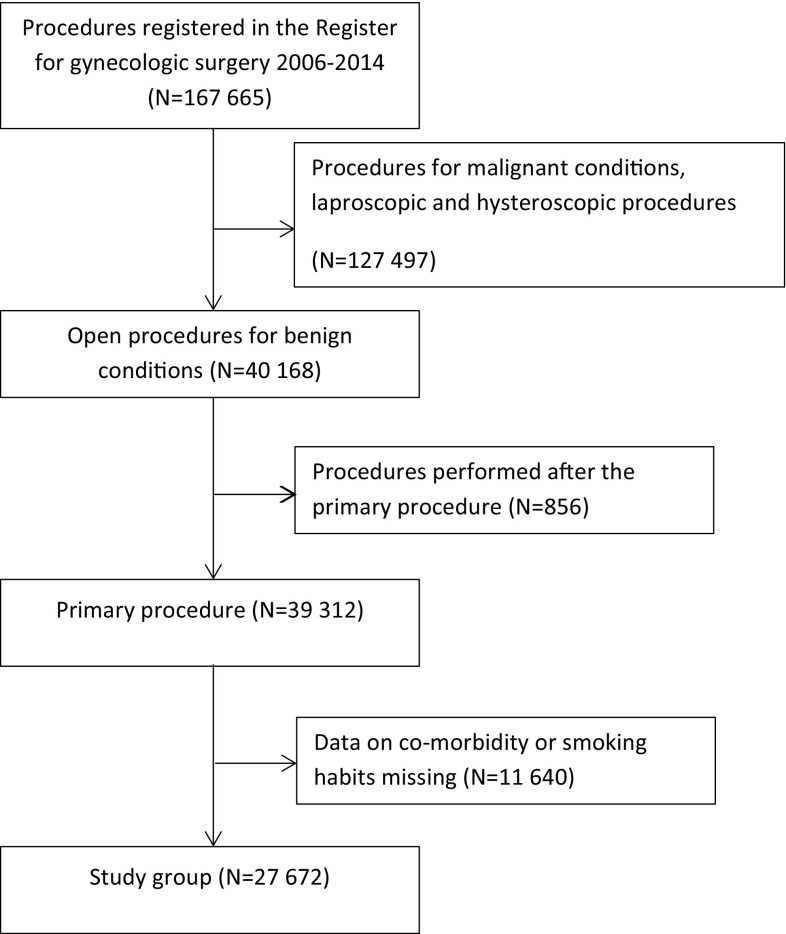
Flow chart GynOp

The Swedish National Quality Register of Gynecological Surgery (GynOp) started in 1997. Today almost all operating Gynecology and Obstetric clinics take part. Additionally, data from The Gynecological Quality Register (used mainly by the Stockholm region) are exported to GynOp. GynOp collects preoperative, intraoperative, and postoperative information on women undergoing gynecological surgery. Obstetric surgeries are not included. Data are collected using doctor's forms, on paper or on-line (since May 2012), and patient questionnaires administered as part of routine medical care. Women are included in the registry before surgery by the operation planner (generally a nurse) whenever a gynecological operation is planned; all registered women can opt out at any time. At inclusion, a preoperative questionnaire is sent to all women for the collection of baseline and demographic data to be completed either on-line or on paper. A follow-up questionnaire is sent to the patient eight weeks postoperatively and records, e.g., complications and the time to return of normal activity. The surgeon confirms or changes the categorization of the patient-reported complications (Fig. 1).

The National Patient Register (NPR) [13] contains physicians’ data from all hospital discharge notes, outpatient care visits, and visits to the emergency department. Both public and private caregivers are required to report data. The register does not include data from general practitioners or other medical professionals.

All open procedures registered in GynOp from January 1, 2006, to December 31, 2014, were identified. Data on incisional hernias were obtained from the National Patient Register (NPR). The linking between the register for gynecological surgery and the NPR was performed using the Swedish Personal Registration Number, which is a ten-digit number unique for each resident [14].

Information about comorbidity was extracted from the GynOp preoperative questionnaire.

### Statistical analyzes

Analyses were performed to identify the impact of each risk factor and to estimate the total incidence of incisional hernias.

Primary outcome was diagnosis or surgery for incisional hernia registered in the NPR after a procedure registered in GynOp. Diagnosis of incisional hernia was defined as a discharge note or outpatient visit with the ICD-10 codes K43.0-K43.9. Surgery for incisional hernia was defined by intervention codes JAD10-JAD87.

The impact of surgical approach on the risk of incisional hernia development was evaluated in a time to event analysis, applying the date of the gynecological procedure as the time of entry in the cohort. Date of death or end of follow-up were treated as censored events. Age, BMI, comorbidity, and type of incision as risk factors for incisional hernias were analyzed with Cox proportional hazard analysis. IBM SPSS Statistics for Windows, Version 20.0. Armonk, NY: IBM Corp was used for the analyzes (Table 1).

**Table 1:** Baseline characteristics

Baseline characteristics	*N* (%)
Mean age, years (standard deviation)	53.1 (13.8)
Mean BMI (standard deviation)	26.8 (6.1)
Smoking habits	
Non-smokers	22 861 (84.0%)
Smokers	4 361 (15.8%)
Self-reported history of chronic disorders	
Pulmonary disease	5 132 (18.5%)
Renal disease	1 088 (3.9%)
Rheumatological conditions	1 387 (5.0%)
Hepatic disease	1 622 (5.9%)
Diabetes	1 618 (5.8%)
Method of approach	
Midline incision	12 858 (46.5%)
Pfannenstiel	10 465 (37.8%)
Cohen	4 349 (15.7%)

## Results

A total of 40,168 open procedures were entered into GynOp between January 1, 2006, and December 31, 2014. 856 women underwent more than one procedure, leaving 39,312 women in the study. Of these, 27,672 responded to the health declaration. These responders constituted the study cohort. Mean follow-up was 3.1 years (Table 2).

**Table 2:** Univariate and multivariate Cox proportional hazard analysis of risk for incisional hernia

Variable	Univariate Cox proportional hazard analysis		Multivariate Cox proportional hazard analysis	
	Hazard ratio (95% confidence interval)	*p*	Hazard ratio (95% confidence interval)	*p*
Age > 60 years	2.04 (1.67–2.51)	< 0.001	1.54 (1.22–1.95)	< 0.001
BMI ≥ 30	4.07 (3.30–5.02)	< 0.001	3.58 (2.88–4.45)	< 0.001
Smoker	1.50 (1.18–1.90)	< 0.001	1.88 (1.45–2.42)	< 0.001
Diabetes	1.82 (1.30–2.56)	0.001		
Kidney disease	1.71 (1.15–2.55)	0.008	1.58 (1.05–2.38)	0.029
Liver disease	1.94 (1.42–2.66)	< 0.001	1.45 (1.04–2.02)	0.030
Rheumatologic disease	1.69 (1.18–2.43)	0.004		
Pulmonary disease	1.61 (1.28–2.02)	< 0.001	1.31 (1.03–1.66)	0.028
Midline incision (Reference Cohen or Pfannenstiel)	2.86 (2.30–3.56)	< 0.001	2.22 (1.73–2.84)	< 0.001

**Fig. 2:**
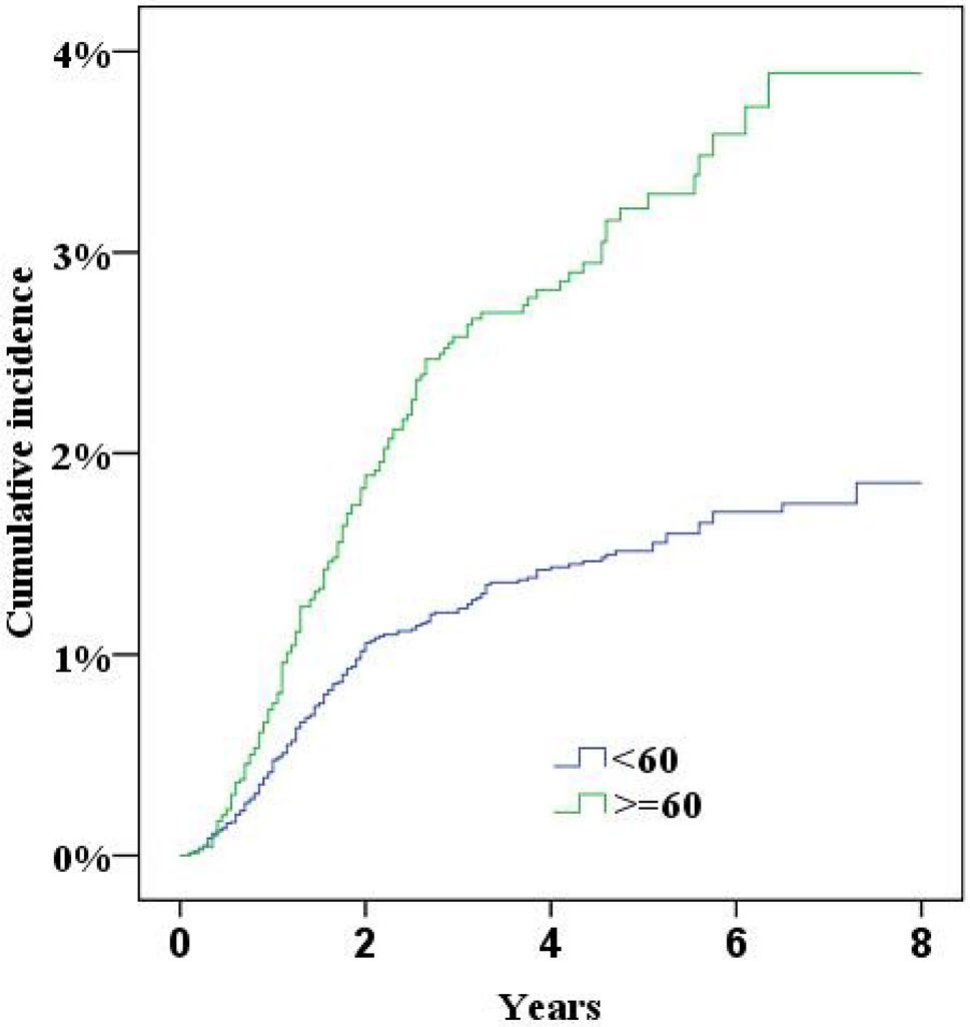
Cumulative incidence of incisional hernia by age (>60 years versus <60 years)

**Fig. 3:**
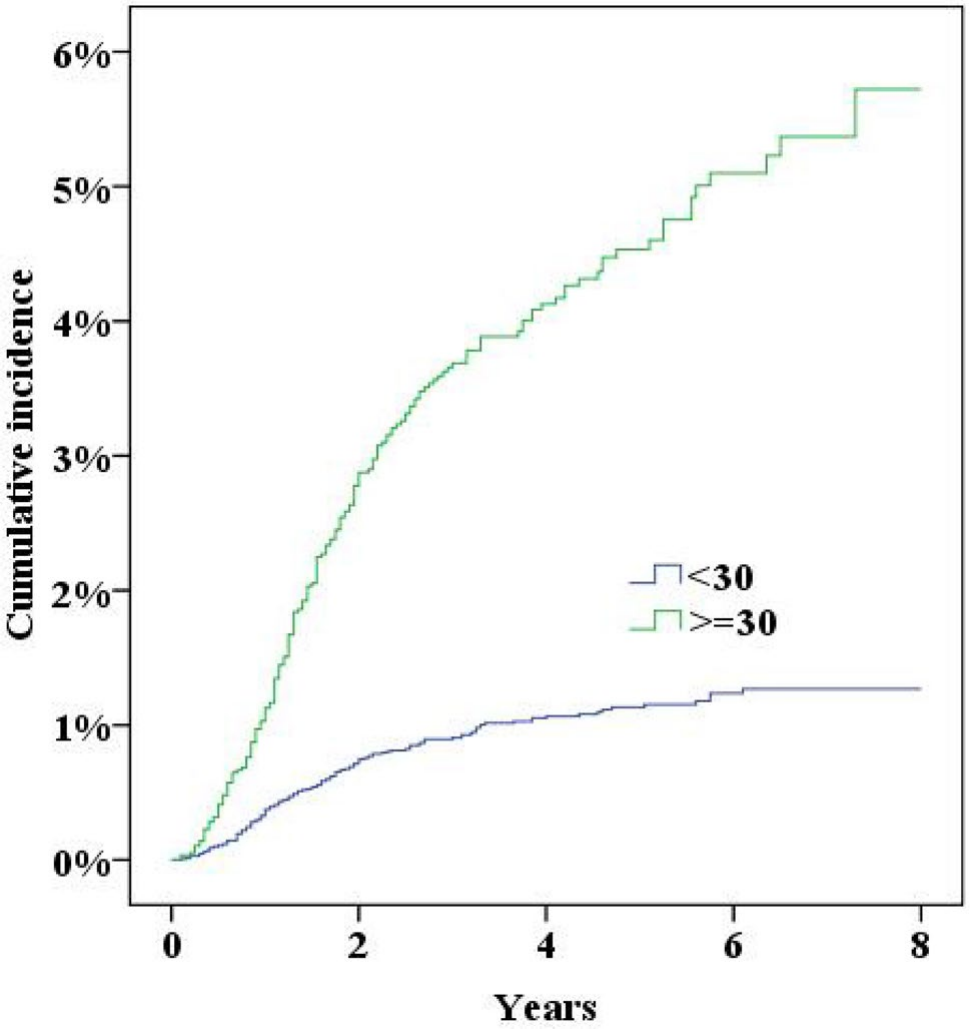
Cumulative incidence of incisional hernia by BMI

**Fig. 4:**
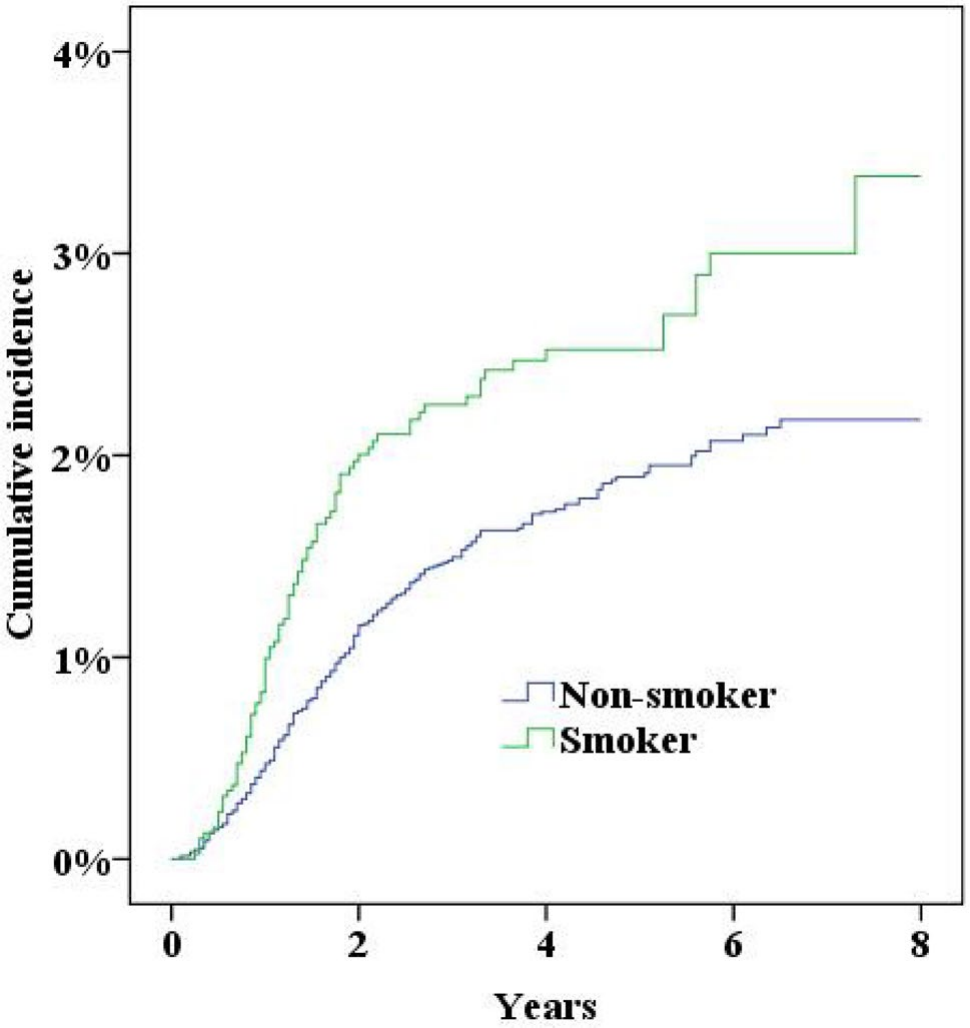
Cumulative incidence of incisional hernia by smoking habits

**Fig. 5:**
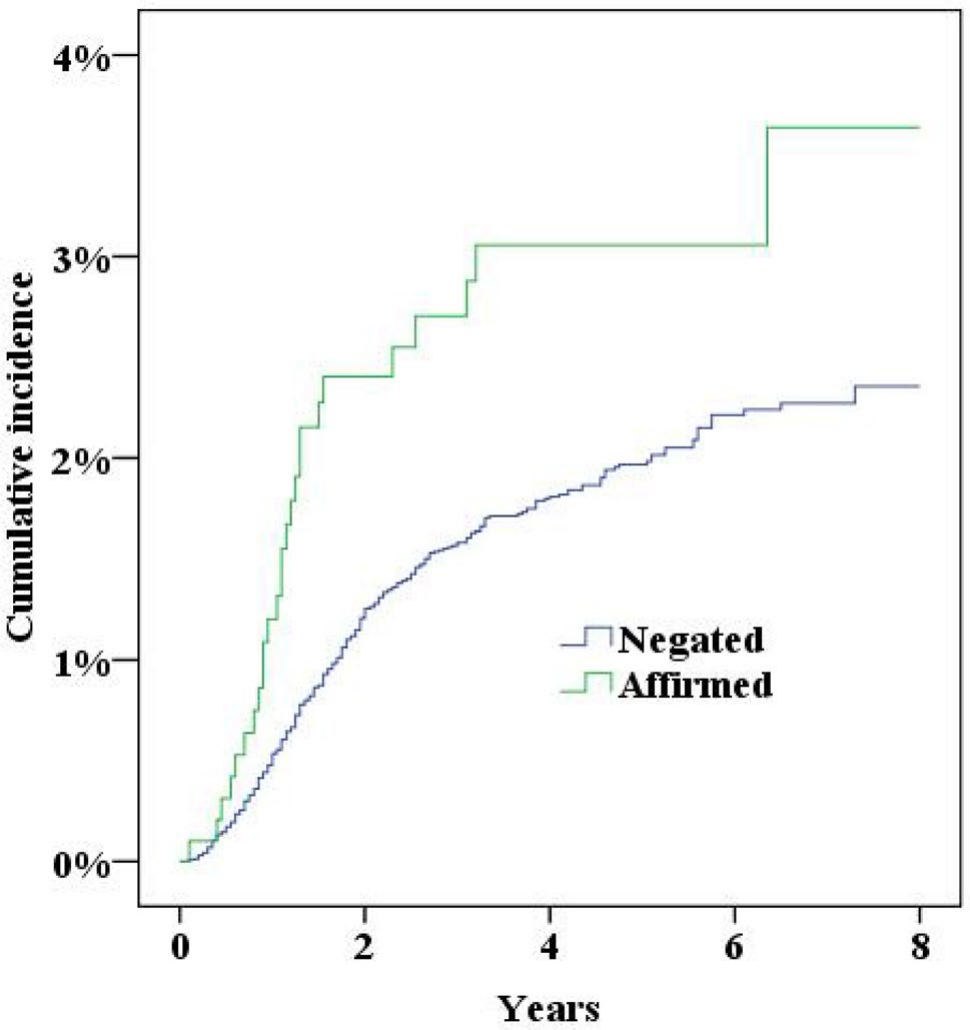
Cumulative incidence of incisional hernia by history of kidney disease

**Fig. 6:**
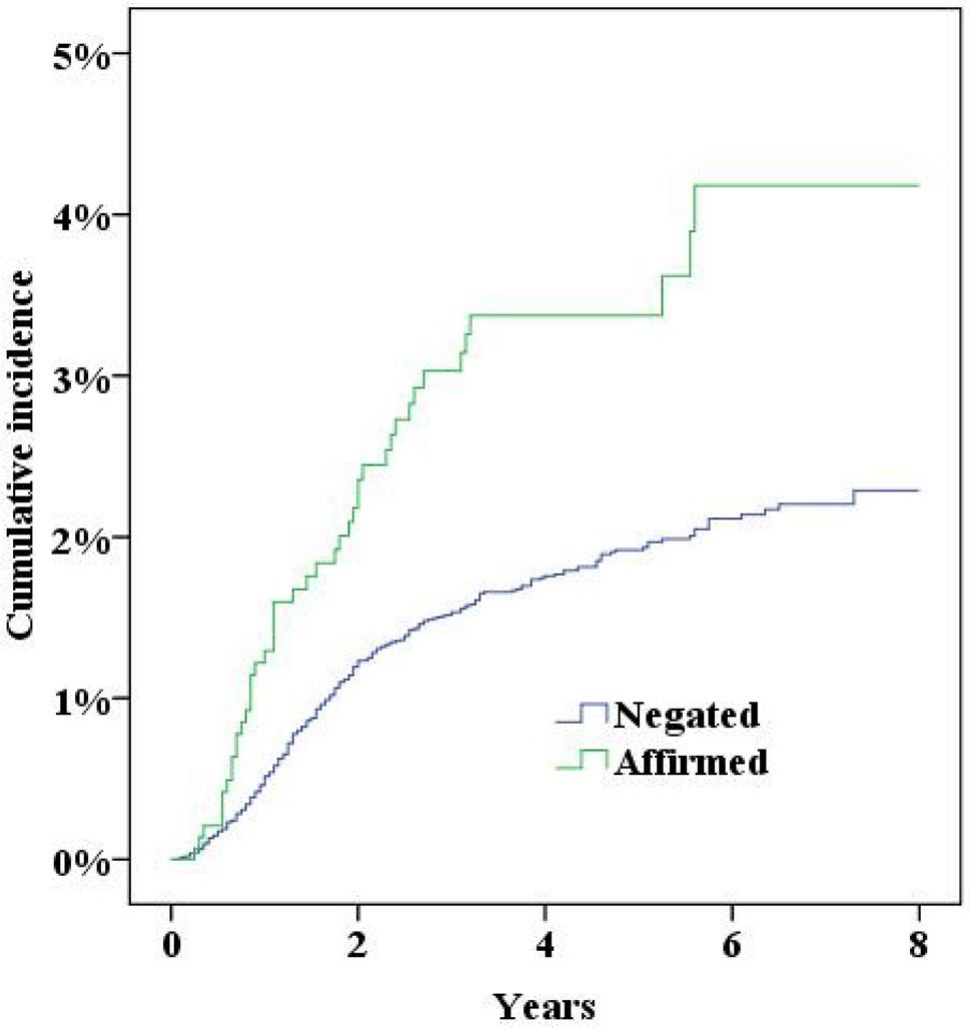
Cumulative incidence of incisional hernia by history of liver disease

**Fig. 7:**
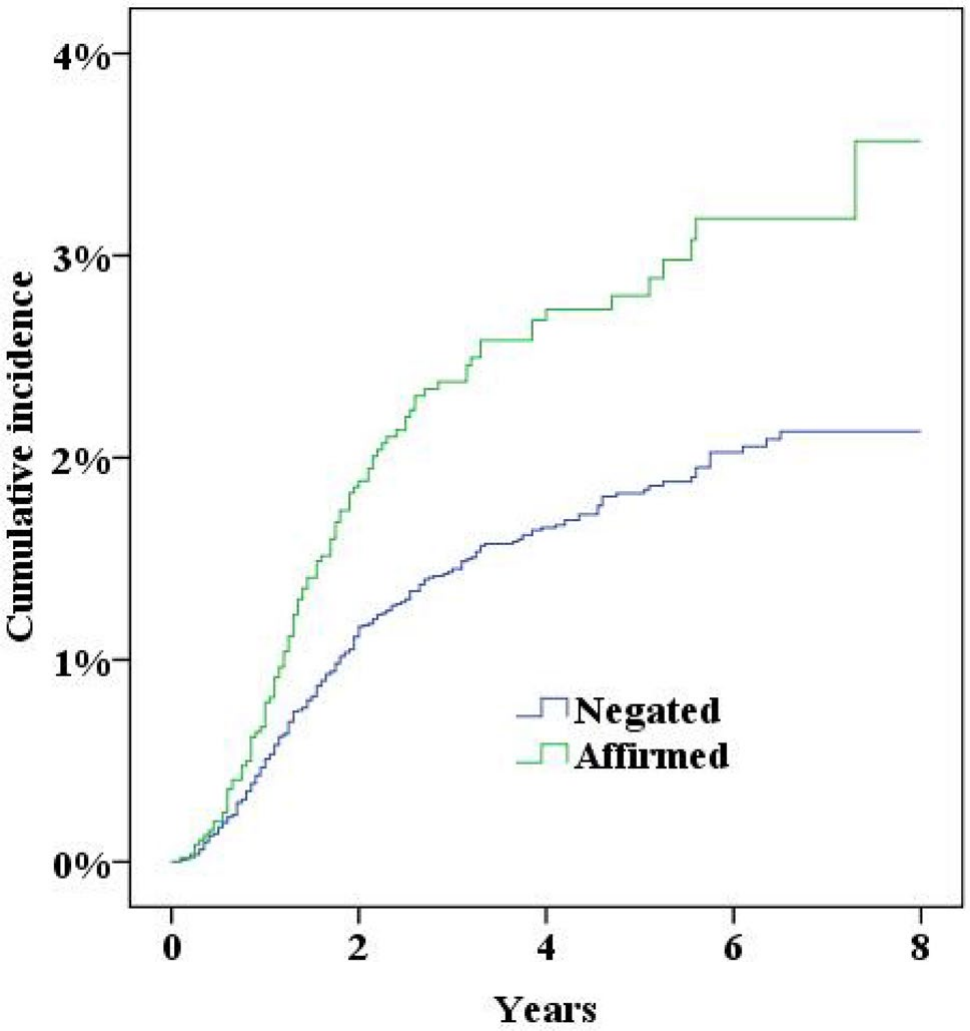
Cumulative incidence of incisional hernia by history of pulmonary disease

**Fig. 8:**
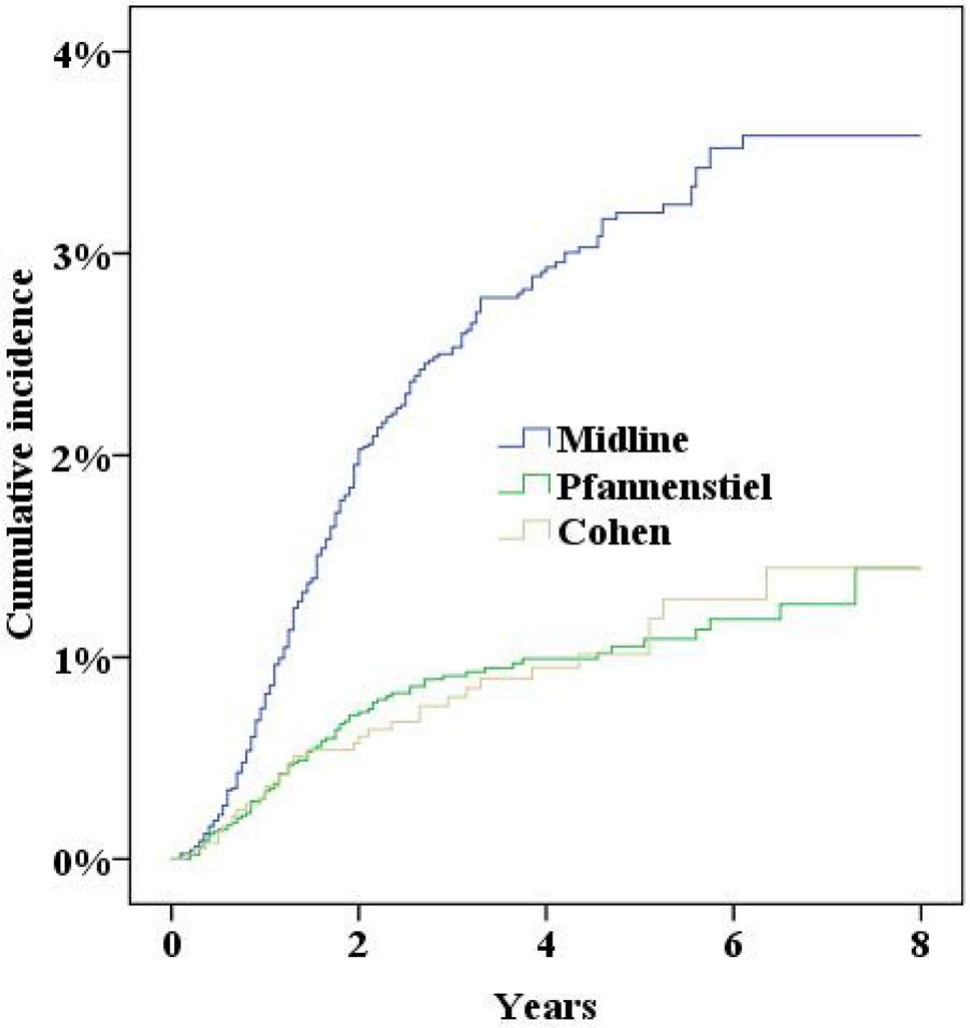
Cumulative incidence of incisional hernia by incision

During the study period the NPR recorded 379 women who had either been diagnosed with incisional hernia or undergone surgery for incisional hernia. Five years after surgery the cumulative incidence of incisional hernias was 2.0% (95% confidence interval 1.8–2.2%). In multivariate Cox proportional hazard analysis a significantly increased risk for incisional hernias was found for a midline incision, obesity (BMI>30), smoking and age (Table 2, all *p* < 0.05; Figs. 2, 3, 4, 5, 6, 7, 8).

## Discussion

The present population-based study shows that the incidence of incisional hernias following gynecological surgery is relatively low. In the Swedish Registry for Ventral Hernias 12.2% of incisional hernia operations were performed after gynecological and obstetrical surgery. A diagnosis or surgery for incisional hernia is a surrogate marker for the actual incidence of incisional hernias and is likely a low estimate. However, these numbers reflect the incidence of clinically relevant incisional hernias. The group of excluded patients (12,496) may hide several incisional hernias.

There are patient categories that face an increased risk of incisional hernia. The variables with the highest impact on development of an incisional hernia were obesity and a midline incision.

Obesity is a great problem in case of surgery. In 2001–2002 Sweden had an obesity (BMI > 30) prevalence of 10% and in 2014 the prevalence had increased to 14% [15]. Obesity is increasing all over the world and it is unfortunate that this group is additionally burdened by an increased incidence of incisional hernias. Israelsson et al. observed that the surgeon tends to use larger tissue bites with greater intervals between stitches on obese patients [4]. Current evidence favors small tissue bites with small intervals using a suture to wound ratio of at least 4:1 [8, 9]. It seems as if we unwittingly increase the risk of incisional hernias in these patients. Furthermore, it can be difficult to palpate incisional hernias in obese patients. A hernia in a Pfannenstiel incision can be even more difficult to palpate and can go unnoticed by the patient.

The Pfannenstiel incision was developed to reduce the incidence of incisional hernias [16] and to this day remains superior to the midline incision in that respect [11]. Unfortunately, the Pfannenstiel and Cohen incisions are not always appropriate, e.g., in extensive oncological surgery where access to the abdomen must be prioritized. The Pfannenstiel incision can also be used successfully in general surgery, e.g., in laparoscopically assisted colectomy for extracting the specimen [17]. However, nerve entrapment is a complication to this incision, especially when extended laterally. This must be taken into consideration when choosing the appropriate incision [10].

Age is also a considerable risk factor in univariate analysis but not so much in multivariate analysis, perhaps due to the fact that comorbidity tends to increase with age.

The reported proportion of smokers in the GynOp Register was 15.8%. Data were missing for 30% of all women. In 2006, 17% of Swedish women smoked daily [18], 11% in 2014 [19]. Therefore, it is reasonable to assume that the self-reported frequency of smoking in the GynOp Register is accurate. We found that smoking was the third most important risk factor in the development of incisional hernias. This is consistent with earlier findings [20]. Preoperative anti-smoking programs have been shown to have beneficial effects on postoperative complications [21]. Many clinics in Sweden have implemented such programs.

We chose to eliminate women who had multiple surgeries from the statistical analysis. This may have reduced the incidence of incisional hernias found in our study. It is known that repeated incisions carry a higher risk of development of an incisional hernia.

The incidence of incisional hernia depends not only on patient- and surgeon-related factors but also on the method of detection. Henriksen et al. reported an incidence of 25.9% on patients who were examined by an experienced surgeon [22], whereas J Nilsson et al. reported an incidence of 30.5% on patients regularly examined with CT scans after surgery for liver metastases of colorectal cancer [23]. The women in our study were not screened for incisional hernia. Therefore, the incidence of incisional hernia is likely underestimated.

Tecce et al. have recently performed a cost-analysis on incisional hernias after hysterectomy. In their material, women with an incisional hernia had fourfold more readmissions, fivefold added hospital length of stay and significantly higher index procedure costs [24].

In conclusion, our study shows that there is much to be gained if patients can cease smoking and lose weight before undergoing abdominal surgery. Many departments specializing in hernia surgery strive to optimize their patients preoperatively through weight loss regimens and anti-smoking programs [25]. This could and should be applied to all patients undergoing elective abdominal surgery. The Pfannenstiel incision results in fewer incisional hernias compared to a midline incision and should be considered whenever possible.

## Abbreviations

GynOp: The Swedish National Quality Register of Gynecological Surgery
NPR: The National Patient Register
